# Atypical presentation of acute pancreatitis in a man with pancreatic insufficiency and cystic fibrosis: a case report

**DOI:** 10.1186/1752-1947-4-275

**Published:** 2010-08-18

**Authors:** Malcolm Turner, Hugh Jackson, Robin Harle, Rob Bohmer, David W Reid

**Affiliations:** 1 Department of Respiratory Medicine, Royal Hobart Hospital, Tasmania, Australia; 2 Department of Gastroenterology, Royal Hobart Hospital, Tasmania, Australia; 3 Department of Radiology, Royal Hobart Hospital, Tasmania, Australia; 4 Department of Surgery, Royal Hobart Hospital, Collins Street, Hobart, 7001, Tasmania, Australia

## Abstract

**Introduction:**

Whether acute pancreatitis can occur in pancreatically insufficient individuals with cystic fibrosis remains a matter of debate.

**Case presentation:**

We describe a case of acute pancreatitis occurring in a 52-year-old Caucasian Australian man with moderately severe cystic fibrosis lung disease and pancreatic insufficiency. An inflammatory mass within the head of his pancreas was confirmed using computed tomography, magnetic resonance imaging and pancreatic biopsy, but serum amylase and lipase remained normal throughout the acute phase of his illness. His symptoms and the pancreatic mass resolved following the insertion of a biliary stent and the introduction of ursodeoxycholic acid.

**Conclusion:**

Our case report highlights the potential for acute pancreatitis to occur in patients with pancreatic insufficiency and cystic fibrosis. We further demonstrate that conventional biochemical markers that are normally assessed to confirm the diagnosis may not be of particular use. As patients with cystic fibrosis survive into their fourth and fifth decades of life, atypical presentations of acute pancreatitis may become more common.

## Introduction

Acute pancreatitis in cystic fibrosis occurs almost exclusively in young patients with pancreatic sufficiency [1]. We describe the case of a 53-year-old man with cystic fibrosis and pancreatic insufficiency who presented with abdominal pain and a diagnosis of acute pancreatitis despite normal amylase and lipase levels in his peripheral blood.

## Case presentation

A 52-year-old Australian Caucasian male with cystic fibrosis was admitted to our hospital with an exacerbation of pulmonary sepsis accompanied by a vague abdominal pain. Abdominal X-ray revealed faecal loading in his caecum and ascending colon with proximal small bowel dilatation consistent with meconium ileus equivalent. His relevant medical history consisted of pancreatic insufficiency and bronchiectasis with moderately severe lung function impairment (FEV_1 _2.18 L/s; 53% predicted). He had multiple hospital admissions over the preceding two years with exacerbations of chronic airway sepsis.

The diagnosis of cystic fibrosis had been made during his early childhood when he presented with failure to thrive, and this had been confirmed with an elevated sweat test and genotyping that revealed him to be a heterozygote for G542X, with the other allele unidentified. G542X is a Class I mutation that results in the complete failure to synthesize functional cystic fibrosis transmembrane conductance regulator (CFTR) and is usually associated with pancreatic insufficiency. On this particular admission, his chest and abdominal symptoms resolved after a course of intravenous antibiotics, fluid replacement, and oral administration of N-acetyl cysteine. His 24-hour fecal fat levels were elevated at 41 grams and his pancreatic enzymes were thus increased.

He presented again after six weeks due to a recurrence of severe abdominal pain, anorexia and weight loss, but without any alteration to his bowel habit. Examination revealed epigastric tenderness and active bowel sounds, but no guarding. Results of respiratory examination were unchanged. On this occasion, abdominal X-ray was normal, as were his full blood count, urea and electrolytes, serum amylase (46 IU/L; NR < 100 IU/L), and liver function tests. However, he continued to complain of severe abdominal pain radiating through his back and lower chest. An abdominal ultrasound demonstrated an increased echogenicity consistent with inspissated secretions in the pancreatic duct and an 8 mm common bile duct with no other abnormalities.

**Figure 1:**
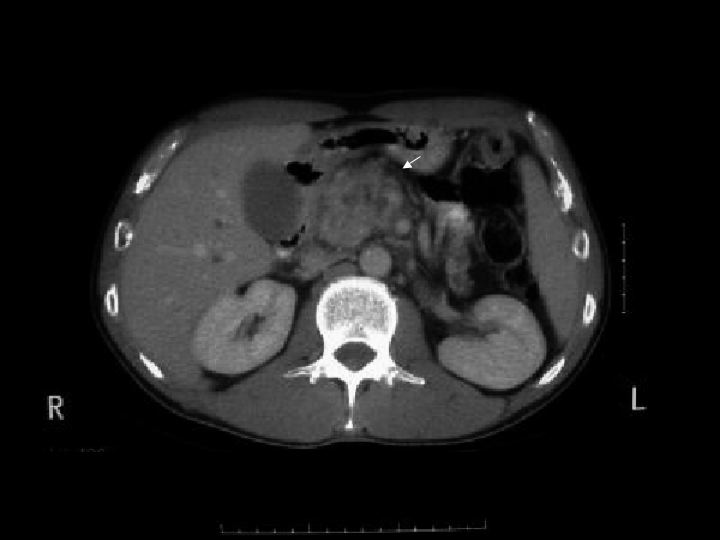
**Computed tomography scan demonstrating mass at the head of the pancreas**.

Two days after admission, our patient became jaundiced. Repeat blood tests demonstrated a cholestatic picture: total protein 66 g/L, albumin 36 g/L, alkaline phosphates (ALP) 809IU/L, alanine transaminase (ALT) 543IU/L, glutamyl transaminase (GGT) 334IU/L, and bilirubin 23 mmol/L. His serum amylase (42IU/L) and lipase (4IU/L; NR: < 10IU/L), as well as urinary lipase (96IU/L, NR: < 500IU/L) levels remained normal. An abdominal computed tomography (CT) scan demonstrated that he had a heterogeneous mass measuring 5×5×5 cm and located at the head of the pancreas with biliary and pancreatic duct dilation (Figure 1). Magnetic resonance cholangiopancreatography confirmed the enlargement of the head of his pancreas with dilated intrahepatic and extrahepatic biliary ducts. These findings were thought to be consistent with either acute pancreatitis or a pancreatic malignancy.

Results of his liver function tests remained abnormal (ALP 1085IU/L, ALT 249IU/L, GGT 383IU/L, and bilirubin 37 μmol/L) and the patient proceeded to endoscopic retrograde cholangiopancreatography, where a narrowing of the common bile duct at the head of the pancreas was identified with more distal anatomical distortion of the entire pancreatic ductal system. A biliary stent was consequently inserted. CT guided biopsy of the pancreatic mass demonstrated reactive pancreatic ductal epithelium with an infiltrate of macrophages and lymphocytes, but no evidence of malignancy was found.

**Figure 2:**
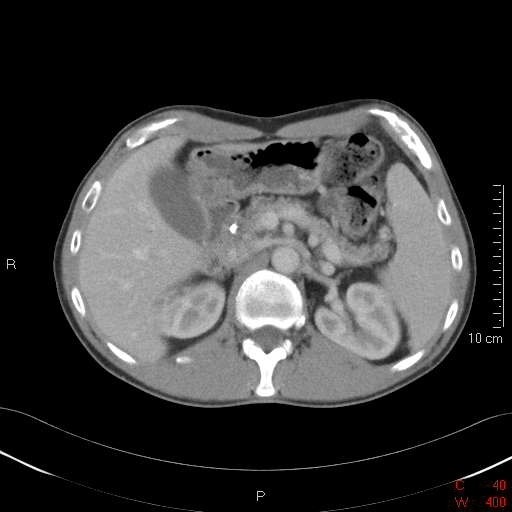
**Computed tomography scan at 18 months showing resolution of the pancreatic inflammatory mass**.

The patient continued to experience abdominal pain and ursodeoxycholic acid was introduced to treat any potential contribution of biliary sludging to cholestasis and also to minimize the risk of stent occlusion. Following stent insertion and commencement of ursodeoxycholic acid, his symptoms and liver function tests slowly improved, and then returned to normal over the next 6 weeks. A repeat CT scan showed a resolution of biliary dilation, but no change in the pancreatic mass was noted. After 18 months he remains well with normal liver function tests and no abdominal pain. A repeat CT scan at this time demonstrated a complete resolution of the pancreatic mass (Figure 2).

## Discussion

Although chronic pancreatitis is common in cystic fibrosis, acute pancreatitis is rare and usually occurs in young patients who are pancreatically sufficient [1,2]. We are unaware of any previous reports of acute pancreatitis occurring in an older adult with pancreatic insufficiency and cystic fibrosis but in the setting of normal amylase and lipase. The few reports available concerning acute pancreatitis in patients with pancreatic insufficiency concern children or young adults and always with raised amylase and lipase blood levels [1,3,4].

In our patient, acute pancreatitis was possibly related to his abnormal pancreatic duct anatomy and the chemical insult of bile constituents causing direct damage to his pancreatic tissue, which was then followed by a local inflammatory response. Despite these proposed mechanisms there was no classical biochemical evidence of pancreatic injury, although imaging confirmed a typical inflammatory mass and gross edema of the pancreas. Clinical improvement was relatively rapid following biliary stenting and the introduction of ursodeoxycholic acid. A repeat CT scan demonstrated a complete resolution of the previous inflammatory mass.

When all other diagnoses have been excluded, the poor diagnostic sensitivity of amylase and lipase in both blood and urine samples have to be considered in older patients with cystic fibrosis who present with abdominal pain.

## Conclusion

As patients with cystic fibrosis survive into their fourth and fifth decades of life, atypical presentations of acute pancreatitis may become more common. Caution needs to be exercised when diagnosing acute pancreatitis in patients with pancreatic insufficiency, as the biochemical parameters normally used may not accurately reflect the disease process.

## Consent

Written informed consent was obtained from the patient for publication of this case report and accompanying images. A copy of the written consent is available for review by the Editor-in-Chief of this journal.

## Competing interests

The authors declare that they have no competing interests.

## Authors' contributions

DR was the consultant physician who cared for the patient at the time of presentation and diagnosis. MT was the junior doctor attached to the respiratory unit at the time. MT identified the unusual nature of the case and wrote the first draft of this case report. DR contributed to the writing of the case report as did his colleagues HJ, RB and RH, all of whom were involved in diagnosing and managing the patient. RH interpreted the radiology results. All authors read and approved the final manuscript.

## References

[B1] De Boeck K Weren M Proesmans M Kerem E Pancreatitis among patients with cystic fibrosis: correlation with pancreatic status and genotype Pediatrics 2005 115 e463 e469 10.1542/peds.2004-1764 15772171

[B2] Walkowiak J Lisowska A Blaszczynski M The changing faces of the exocrine pancreas in cystic fibrosis: pancreatic sufficiency, pancreatitis and genotype Eur J Gastroenterol Hepatol 2008 20 157 160 10.1097/MEG.0b013e3282f36d16 18301292

[B3] Maiz L Kirchschläger E Suárez L Escobar H Acute pancreatitis in a patient with cystic fibrosis and pancreatic insufficiency Rev Esp Enferm Dig 1996 88 581 582 8962768

[B4] Moreno E Gonzalez Ibaoez J Aguirre Rico P Selas Vorwald P Santoyo J Santoyo Gomez R Sanz Seoane J Gonzalez Figueroa J Andollo Sciadini M Recurrent acute pancreatitis as a complication of cystic fibrosis: report of one case treated surgically Ann Ital Chir 1991 62 345 347 1768003

